# Safety, cost and environmental impact of reprocessing low and moderate risk single-use medical devices: a systematic review

**DOI:** 10.3205/dgkh000617

**Published:** 2026-01-26

**Authors:** Niamh McGrath, Catherine Waldron, Ailish Farragher, Áine Teahan, Leila Keshtkar, Julie Polisena, Francesco Tessarolo

**Affiliations:** 1 Evidence Centre, Health Information and Evidence Directorate, Health Research Board, Dublin, Ireland; 2 Health Technology Assessment Division, International Federation of Medical and Biomedical Engineering, Ottawa, Canada; 3 Department of Industrial Engineering, University of Trento, Trento, Italy; 4 Healthcare Research and Innovation Program, Bruno Kessler Foundation, Trento, Italy

**Keywords:** reprocessing single-use devices class I, reprocessing single-use devices class II, endoscopic devices, laparoscopic devices, ophthalmic devices, surgical instruments, external fixators, stockings, compression sleeves, pulse oximeters, patient safety, costs, environmental impacts

## Abstract

**Objectives:**

Estimate the safety, financial and environmental impacts of reprocessing low and moderate risk single-use medical devices (SuMDs).

**Methods:**

Systematic review (PROSPERO ID: CRD42022365642) of primary studies of patients receiving reprocessed low and moderate risk SuMDs (non-critical and semi-critical medical devices a and b) versus first use of otherwise identical SuMDs. Items were sourced via database and supplemental searching. Results were reported by device risk class, included studies were quality appraised, and primary outcomes: direct patient safety; direct and indirect financial costs; environmental impacts, were Grade of Recommendation, Assessment, Development and Evaluation (GRADE) assessed following narrative synthesis.

**Results:**

Ten studies examined 10 devices across three categories of risk class I devices: external fixator devices (n=3 studies), compression sleeves (n=2), and pulse oximeters (n=1) and three categories of risk class II devices: ophthalmic devices (n=1), surgical instruments for grasping and cutting (n=1) and endoscopic and laparoscopic devices (n=5 studies, 5 devices).

There were no significant differences in the odds of primary safety outcomes across the two device types contributing data. The only study contributing primary financial impact data reported no statistically significant difference in savings for new versus reprocessed devices (p=0.340). Reprocessing reduced global warming (n=2 studies) and increased human health impacts (n=1) across the four device types contributing data. The certainty of safety and cost evidence was very low.

**Conclusions:**

Safety monitoring systems where SuMD reprocessing is permitted are required. Reprocessing costs should be estimated using appropriate methodologies and research is needed to ensure that life cycle assessment study designs can be better utilised to inform decision-making.

## Zusammenfassung

**Zielsetzung:**

Abschätzung der Sicherheit, finanzieller und Umweltauswirkungen der Wiederaufbereitung von Medizinprodukten für den Einmalgebrauch mit geringem und mittlerem Risiko (SuMDs).

**Methode:**

Systematische Überprüfung (PROSPERO ID: CRD42022365642) von Primärstudien über Patienten, die wiederaufbereitete SuMDs mit niedrigem und mittlerem Risiko (unkritisch und semikritisch a and b) erhalten haben, im Vergleich zur Erstverwendung von ansonsten identischen SuMDs. Die Artikel wurden über eine Datenbank- und Ergänzungssuche beschafft. Die Ergebnisse wurden nach Geräte-Risikoklasse angegeben, die Qualität der eingeschlossenen Studien wurde bewertet, und die primären Ergebnisse – direkte Patientensicherheit, direkte und indirekte finanzielle Kosten sowie Umweltauswirkungen - wurden nach einer narrativen Synthese nach dem GRADE-System (Grade of Recommendation, Assessment, Development and Evaluation) bewertet.

**Ergebnisse:**

Zehn Studien untersuchten 10 Produkte aus drei Kategorien der Risikoklasse I: externe Fixateure (n=3 Studien), Kompressionsmanschetten (n=2) und Pulsoximeter (n=1) und drei Kategorien von Produkten der Risikoklasse II: ophthalmologische Produkte (n=1), chirurgische Instrumente zum Greifen und Schneiden (n=1) und endoskopische und laparoskopische Produkte (n=5 Studien, 5 Produkte).

Zwischen den beiden Produkttypen gab es keine signifikanten Unterschiede in der Wahrscheinlichkeit der primären Sicherheitsauswirkungen. Die einzige Studie, die Daten zu den primären finanziellen Auswirkungen beisteuerte, meldete keinen statistisch signifikanten Unterschied bei den Einsparungen zwischen neuen und wiederaufbereiteten Medizinprodukten. Die Wiederaufbereitung reduzierte die globale Erwärmung (n=2 Studien) und erhöhte die Auswirkungen auf die menschliche Gesundheit (n=1) bei allen vier Produkttypen. Die Evidenz in Bezug auf Sicherheit und Kosten war sehr gering.

**Schlussfolgerungen:**

Sofern die Wiederaufbereitung von SuMDs erlaubt ist, sind Sicherheitsüberwachungssysteme erforderlich. Die Wiederaufbereitungskosten sollten mit Hilfe geeigneter Methoden geschätzt werden, und es sind Forschungsarbeiten erforderlich, um sicherzustellen, dass Studien zur Lebenszyklusbewertung besser zur Entscheidungsfindung genutzt werden können.

## Introduction

Single-use medical devices (SuMDs) are intended by their manufacturers to be used once and then discarded. In an effort to mitigate the costs [1] and environmental footprint [2] of health care, SuMDs reprocessing is practiced globally [1], [3], [4]. Reprocessing, a process carried out on a used device to allow its safe reuse, involves cleaning, disinfection, sterilisation and related procedures, as well as testing and restoring the safety and performance of the used device [3]. There are no requirements for manufacturers to prove that a device cannot be reprocessed [1] and reprocessing industry stakeholders in Europe have estimated that 16% of devices labelled as being for “single-use” may technically be safe and effective to reprocess for a limited number of times [4]. However, adverse events associated with reprocessing have been reported [1].

Regulating SuMD reprocessing could reduce the risk of reprocessing related adverse events. Regulation likely reduces the volume of in-house (health facility) reprocessing due to the high cost and staff education and training implications of implementing regulatory standards [2]. In turn, it may reduce the number of SuMDs reprocessed, as seen in Germany and Australia [1]. Regulation may also inform the types of SuMDs reprocessed. For example, since 2000, the FDA has approved the reprocessing of over 100 SuMDs in the USA [5], with lower risk SuMDs (i.e. those which do not come into contact with the bloodstream or other sterile areas of the body), the most frequently reprocessed there [5]. 

That regulated reprocessing reduces the volume of in-house reprocessing raises questions about the cost-effectiveness of SUMD reprocessing under regulated conditions. Generally, greater financial savings would be expected from high-risk devices compared to low and moderate risk devices as their more complex designs make them more expensive to produce [6]. The European reprocessing industry [4] and available systematic review evidence [7], [8] are consistent in reporting that savings could differ by device. Potential saving estimates are frequently cited as 90% when reprocessing is undertaken at a health facility and 50% when reprocessing is undertaken by a third-party reprocessing company [4]. To date, the scientific literature has been unable to confirm these estimates whereby available systematic reviews could not establish the cost-effectiveness of reusing SuMDs due to an inconclusive evidence base and a paucity of high-quality, appropriately designed studies [7], [8]. Life cycle assessment studies, which examine the environmental impact of a medical device from its development to disposal, demonstrate that SuMDs typically result in higher petrochemical use and global greenhouse gas emissions compared with reusable alternatives [9], [10]. However, it is not yet known whether reprocessing and reusing these SuMDs is more environmentally beneficial than their one-time use and subsequent disposal.

## Objectives

As part of efforts to keep the EU Medical Device Regulation (MDR) legislative decision adopted by Ireland under review, the Health Research Board completed an evidence review requested by the Department of Health in Ireland on the safety, financial costs and environmental impacts of reprocessing SuMDs. The current article presents the findings of the systematic review of risk class I and risk class IIa and IIb devices. 

The aims of this review are to:

- Identify the risk class I and II SuMDs safe to reprocess in line with the 2017 EU medical device regulation and other related approaches, and
- Synthesise the safety, financial and environmental consequences of risk class I and II SuMDs reprocessing in line with the 2017 EU medical device regulation and other related approaches as well as any differences across SuMDs types.

## Methods

### Review design

A systematic review was conducted [11] and reported according to the Preferred Reporting Items for Systematic Reviews and Meta-Analyses (PRISMA) criteria [12], [13]. Where appropriate, procedures were consistent with guidance on systematic reviews with cost and cost-effectiveness outcomes [14]. The original study protocol was registered on the International Prospective Register of Systematic Reviews (PROSPERO) (ID: CRD42022365642). In this article, we present the results of human studies of risk class I and II SuMDs only. A report of risk class III devices has been published already [15].

### Literature search strategy

We searched the following bibliographic databases from their inception: EMBASE, MEDLINE (Ovid platform), Dimensions, and the Cochrane Library. The peer-reviewed search strategy, using National Library of Medicine’s medical subject headings (MeSH), and keywords, centred on five concepts: single-use medical devices; reprocessing; safety and/or adverse outcomes; cost and cost-effectiveness; and environmental impacts. 

Supplementary (i.e. reference and citation checking of included studies and relevant systematic reviews), and grey literature (i.e. government and regulatory authority websites; trial registers; Google.com and Google Scholar search engines [results 1–200] searches were also performed. 

We limited the search to English and German language documents, owing to Germany’s experience in SuMDs reprocessing. Searches were undertaken between 25 July and 23 September 2022 and updated in January 2024. The search strategy is available in Attachment 1. 

### Eligibility criteria

The eligibility criteria were defined using the Population Intervention Comparison Outcomes Study design (PICOS) framework (Table 1 (Tab. 1)). SuMDs included devices and purpose-built components thereof exposed to human cells, bacteria and/or viruses. Reprocessing was defined using European legislation as “a process carried out on a used device in order to allow its safe reuse, including cleaning, disinfection, necessary sterilisation and related procedures, as well as testing and restoring the technical and functional safety of the used device” [16], with a similar definition employed in medical device research [17]. To ensure health system comparability, primary studies of any healthcare facility using reprocessed SuMDs in Organisation for Economic Co-operation and Development (OECD) or EU member states only were eligible. Studies must have included at least one type of primary outcome of interest and compared outcomes with first use of the same SuMD. We did not include systematic review studies as we were uncertain that the evidence included in the reviews would reflect reprocessing as defined in our study [7], [8]. 

### Article selection

Following deduplication in EndNote, two of three possible screeners (NMG, LK, CW) screened each item in EPPI Reviewer at title and abstract and again at full-text screening stages. Disagreements were resolved by consensus at both stages. Where individual study eligibility was unclear due to missing information at full text screening stage, study authors were contacted to seek clarification. If study authors did not respond within two weeks after the initial email, and one week after a reminder email, the study was excluded. 

### Data extraction and outcome selection

Study data were extracted independently by two of four reviewers (NMG, CW, LK, ÁT) into study design specific extraction forms in Microsoft Word and subsequently agreed by the two reviewers. Third-party arbitration was used to resolve disagreements. During extraction, devices were classified as risk class I, IIa or IIb, using Medical Device Coordination Group guidance [18]. The system, created to support implementation of the 2017 EU Medical Device Regulation [3], [16], is similar to the Spaulding Classification System employed in the USA [19] and considers more factors in the assignment of risk classes [18]. 

Safety and cost outcomes were selected for extraction by the review team based on their prevalence across device-specific studies, objective measurement, transparency of reporting, and cost sources (Attachment 2). Primary outcomes were those which:

- Directly impacted patient safety (e.g. complications, functionality loss),
- accounted for both direct and indirect reprocessing costs (e.g. implementing reprocessing or due to infections), and
- directly adversely impact the environment (e.g. global warming potentials).

Secondary outcomes were those which:

- Indirectly impacted patient safety (e.g. procedure time),
- accounted for direct reprocessing costs only, and
- estimated environment-related human health impacts (e.g. toxicological effects of a process).

### Quality assessment

Two reviewers independently assessed the quality of the studies included, with disagreements resolved by consensus. Adapted versions of the 27-item Downs and Black [20] and 19-item Consensus Health Economic Criteria list (CHEC-list) [21] were employed to quality appraise randomised and non-randomised studies and economic study designs. In the absence of a critical appraisal tool for life cycle assessment (LCA) study designs, we employed a transparency checklist proposed by Keil et al. [22]. The checklist was based on German Institute for Standardization (Deutsches Institut für Normung; DIN) and International Organization for Standardization (ISO) standards DIN ISO 14040 and DIN ISO 14044. In keeping with the approach adopted by Keil et al., we report the proportion of items individual study authors report information on. Details of the adaptations made to the quality appraisal tools are reported in Attachment 3 .

### Data analysis and synthesis

We completed an assessment of the feasibility of meta-analysis for each outcome following published guidance [23], [24] (Attachment 4). Based on the results, a narrative synthesis using structured reporting of effects was completed, calculating a standardised effect measure for safety outcomes; odds ratios for categorical outcomes and mean differences for continuous outcomes, and reporting of the number of observed events in the total population for categorical outcomes and the mean/median with standard deviations (SDs) for continuous outcomes [24]. 

### Grading of recommendations, assessment, development and evaluations

The GRADE system was employed to determine a level of confidence, ranging from very low to high, in individual review outcomes based on the contributing primary studies [25]. In line with best practice, we only applied GRADE assessments to primary review outcomes [25]. We did not apply GRADE to environmental outcomes. 

## Results

### Search results and included studies

**Figure 1:**
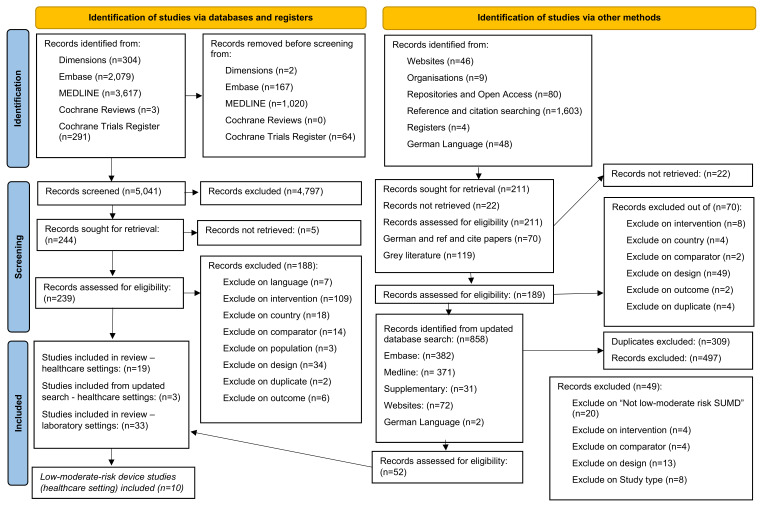
PRISMA flow diagram of search results

Details of the search results and the PRISMA flow diagram are reported in Figure 1 (Fig. 1). We identified 10 studies [26], [27], [28], [29], [30], [31], [32], [33], [34], [35] examining three types of risk class I device: external fixator devices (n=3 studies, 1 device) [26], [27], [28]; compression sleeves (n=2 studies, 1 device) [29], [30]; and pulse oximeters (n=1 study, 1 device) [29], and three types of risk class II device: ophthalmic devices (n=1 study, 1 device) [31]; surgical instruments for grasping and cutting (n=1 study, 1 device) [29]; and endoscopic and laparoscopic devices (n=5 studies, 5 devices) [29], [32], [33], [34], [35]. 

### Characteristics of included studies

The characteristics of included studies are reported in Table 2 (Tab. 2). The studies were undertaken in the USA (n=8) [26], [27], [28], [29], [30], [31], [32], [34] and Europe (n=2; Portugal and Croatia) [33], [35]. Study designs were classified as: randomised controlled trials (n=2 studies) [28], [35]; observational (n=4 studies) [26], [31], [32], [33]; costing (n=2 studies) [27], [34]; and life cycle assessment (n=2 studies) [29], [30]. 

Safety outcome data were available for external fixator devices; ophthalmic devices; and endoscopic and laparoscopic devices. Cost outcomes were available for all device types, except for ophthalmic devices. Environmental outcomes were available for: compression sleeves; pulse oximeters; surgical instruments for grasping and cutting; and four endoscopic and laparoscopic devices. 

Devices were reprocessed at hospital sterilisation departments in one (33%) external fixator device study [26], the (100%) ophthalmic device study [31], and two (20%) endoscopic and laparoscopic device studies (n=2 devices) [34], [35]. Otherwise, reprocessing was undertaken by an external reprocessing company or the original device manufacturer. Most studies reported compliance with FDA reprocessing requirements (n=5 studies; 50%) and others followed local hospital or national policies (n=3 studies; 30%) or research team criteria (n=2 studies; 20%). The number of reprocessing cycles of the same device ranged from 1 [32], [33], [35] to 9 [34].

### Safety outcomes

Studies providing safety data were of poor/low to excellent quality based on the Downs and Black checklist (Table 2 (Tab. 2) and Attachment 3).

#### External fixator devices (risk class I)

No external fixator device safety outcomes were feasible for meta-analysis and were reported narratively (Attachment 4; Table 3 (Tab. 3)). Dirschl and Smith put devices through up to two reprocessing cycles and reported the outcomes for the overall reuse programme only [26]. The overlapping confidence intervals (CIs) indicated similar odds of infection between once-reprocessed devices and new SuMDs across studies (Table 3 (Tab. 3)). Sung et al. also reported no difference in the rate of loss of device fixation or loosening of device components between reused devices and new SuMDs [28]. 

#### Ophthalmic devices (risk class I)

One study contributing data on phaco needle tip reprocessing and reuse safety [31] reported no intraoperative problems or postoperative complications attributable to phaco needle tips in the single-use or reused device groups. The authors reported that there was no association between phacoemulsification time and the number of device reuses (up to five uses), but did not report statistical data to support this statement [31].

#### Endoscopic and laparoscopic devices (risk class IIa)

Endoscopic and laparoscopic device safety outcomes were not feasible for meta-analysis due to inconsistent statistical outcome reporting and heterogeneity in author definitions of complication outcomes (Attachment 2). Four outcomes: reoperations; post-operative complications, procedure time and duration of hospital stay were available for laparoscopic sealer/divider [32], ultrasonic scalpel/shears/scissors [33], [35], and linear suture machine [33] devices. The odds of reoperations [32], reoperations and postoperative complications [33], and postoperative complications [35] were consistently reduced in the reused group compared with the SuMD group, but differences did not reach statistical significance. There were no statistically significant differences in procedure time between procedures employing new and those employing once-reprocessed devices, but conflicting results were reported for duration of hospital stay (Table 3 (Tab. 3)).

### Cost outcomes

Studies providing data on cost outcomes were of low to good quality or reported 68% of items on a transparency reporting checklist (Table 2 (Tab. 2) and Attachment 3). 

#### External fixator devices (risk class I)

Two studies – Horwitz et al. [27] and Sung et al. [28] – reported on one direct cost outcome: savings incurred by the hospital during the study period. Both studies captured US dollar (US$) costs during a similar time frame (between 2001 and 2005) and assumed that a similar proportion (between 75% and 80%) of devices could pass reprocessing requirements. Horwitz et al. reported that reuse of reprocessed external components resulted in savings of 25% and savings of 21% when accounting for the cost of internal components of fixation devices [27]. Sung et al. reported savings of 45% did not account for the device reuse rate [28] (Table 3 (Tab. 3)). 

#### Deep vein thrombosis compression sleeves (risk class I)

Of the seven devices examined in Unger and Landis’s study, deep vein thrombosis compression sleeves had the highest potential for device life cycle cost savings [29] with incremental savings diminishing with each additional reprocessing cycle, up to five cycles (Table 3 (Tab. 3)). 

#### Pulse oximeter (risk class I)

Pulse oximeter device reprocessing resulted in device life cycle cost savings with diminishing incremental savings with each additional reprocessing cycle, up to five cycles [29] (Table 3 (Tab. 3)).

#### Surgical instruments for grasping and cutting (risk class IIa)

Arthroscopic shaver device reprocessing resulted in device life cycle cost savings with diminishing incremental savings after each reprocessing cycle [29] (Table 3 (Tab. 3)).

#### Endoscopic and laparoscopic devices (risk class IIa)

Three of the four studies captured costs in US$ [29], [32], [34], and three studies estimated costs during a similar time frame (2013–2015) [29], [32], [33]. Three studies examined direct, procedure-related costs [32], [33], [34]. One study reported a significant decrease (US$282) in cost in the reprocessed compared with single-use group (*p*=0.028) [32]. When accounting for both direct and indirect costs, savings were sustained but were no longer statistically significant (*p*=0.340) [32]. Two studies reported annual hospital cost savings in the reprocessed group compared with the single-use group: US$65,961 when 222 devices were reused for an average of 2.4 times [34], €14,623.61 based on reuse of 193 linear suture machines compared with purchasing 178 new linear suture machines, and €75,932.55 based on reuse of 418 ultrasonic scalpel/shears/scissors and purchase of 285 new ultrasonic scalpel/shears/scissors over the study period [33]. One study [29] reported small incremental device life cycle related cost savings with each additional reprocessing cycle for each of the four endoscopic and laparoscopic device examined (Table 3 (Tab. 3)).

### Environmental outcomes

Environmental impact outcome data were available across two studies for compression sleeve devices [29], [30] and from one study for pulse oximeter, surgical instruments for grasping and cutting, and endoscopic and laparoscopic devices [29]. The functional unit in the Lichtnegger et al. study [30] was five uses of an intermittent pneumatic compression sleeve whereas, in the Unger and Landis report [29], it was annual use of seven single-use devices, used up to 5 times in a single hospital. Therefore, results are reported together across devices in the Under and Landis report. Both studies providing data on environmental outcomes [29], [30] reported 68%–81% of items on a transparency reporting checklist (Table 2 (Tab. 2) and Attachment 3). 

In their one-to-one device comparison (i.e. exclusion of annual device use), Unger and Landis [29] reported the following relative global warming and human health outcomes: carcinogenic, non-carcinogenic and respiratory impacts. Of the devices studied, the compression sleeve had the highest global warming and non-carcinogenic impacts, and the laparoscopic sealer/divider had the highest carcinogenic and respiratory impacts [29]. Device impacts, normalised to the device with the highest impact for each outcome, are reported in Table 4 (Tab. 4). When accounting for annual use of all seven devices using median/mean reprocessing lifecycle inventory inputs, reprocessing resulted in a reduced and normalised global warming impact with each additional reprocessing cycle compared to single device use. When accounting for annual use of all seven devices using median/mean reprocessing lifecycle inventory inputs, reprocessing resulted in increased normalised carcinogenic, non-carcinogenic and respiratory impacts with each additional reprocessing cycle compared to single device use (Table 4 (Tab. 4)). In the study by Lichtnegger et al. [30], results related to the product contribution to ecological footprint of a person across the 4 impact domains. The authors reported reduced environmental contribution of the reused versus the new devices across all impact domains (Table 4 (Tab. 4)). They further quantified the reduction in global warming potential (kg CO2_eq_) of 7.0 for single use to 4.2 for treatment of five patients using reprocessed devices. 

### Grading of recommendations, assessment, development and evaluations rating

Eligible outcomes for the GRADE process were available for external fixator and endoscopic and laparoscopic devices. Specifically, the GRADE process was applied to four outcomes; pin tract infections (external fixator devices), reoperations (external fixator devices), post operative complications, including reoperations (endoscopic and laparoscopic devices) and total hospitalisation costs (endoscopic and laparoscopic devices). For all outcomes, the a priori rating was “low”, because most of the evidence for each of the four primary outcomes was derived from observational studies. Each outcome received at least one downgrade across two or more domains. When downgrades were applied, all outcomes received a final rating of very low certainty in the evidence. A summary table of judgements are provided in Table 5 (Tab. 5) with explanations provided in Attachment 5.

## Discussion

This study synthesises the available published evidence on SuMDs reprocessing across low and moderate risk devices, incorporating outcomes central to SuMD reprocessing debate (i.e. safety, economic and environmental considerations) [1], [2]. We identified 10 SuMDs across six types of risk class I and risk class II devices. 

Apart from divergent findings on differences in duration of hospital stay post-procedure, we found no additional adverse safety events following SuMD reprocessing. This finding aligns with the results of a 2008 FDA audit of reprocessing approved SuMDs which reported no additional adverse safety effects for external fixation devices, laparoscopic instruments, compression sleeves, pulse oximeters, and arthroscopic accessories [5]. As with previous similar studies [7], [8] we could not estimate the cost-effectiveness of SuMD reprocessing from the available data. Consistent with Hailey et al. [8], this report demonstrates that indirect costs of SuMD reprocessing significantly reduces cost savings. 

Based on the results of the GRADE assessment, we have very low confidence that the results for primary review safety and cost outcomes would be replicated in future studies. There was some evidence of positive and negative environmental impacts of SuMDs reprocessing, and of different environmental impacts by device.

### Future research

The results of this systematic review point toward a need for careful monitoring of the safety of risk class I and risk class IIa SuMDs reprocessing where the practice is permitted under legislation. 

Furthermore, the findings underscore a need to estimate reprocessing costs using appropriate methodologies (e.g. economic evaluation studies), which is consistent with previous systematic reviews calling for additional cost-effectiveness research [7], [8], [36]. Specifically, future primary evaluation studies should consider procurement costs, reprocessing costs, care delivery costs, reprocessing implementation costs, and potential differences in device reprocessing lifespan. For instance, neither of the included external fixator device studies accounted for indirect hospitalisation costs in spite of observed non-statistically significant increases in some adverse safety outcomes [27], [28]. The high cost implications for health facilities of implementing regulatory SuMD reprocessing standards has also been noted [2]. By making scientific and social value judgement more explicit, full economic evaluations enable accountability and transparency about the health care delivery choices made on behalf of others [37]. As a result, full economic evaluation studies could assist EU member states in informing legislative options set out in the 2017 EU Medical Device Regulation, as well as other countries considering the question of SuMDs reprocessing. 

To our knowledge, ours is the first systematic review to capture environmental impacts of SuMDs reprocessing. The results highlight areas for methodological development in life cycle assessment research applied to healthcare and health services evidence synthesis to best utilise them to inform decision-making. In 2021, McGinnis et al. [38] described life cycle assessment studies applied to medical products and processes as *“relatively new”*. Specifically, available reporting checklists research should be validated, quality appraisal tools and reporting guidelines should be developed, as well as supports for applying the GRADE criteria to outcome data. In undertaking this methodological development work, life cycle assessment studies will be able to undergo all critical stages of a systematic review and will be more effective in informing decision-making in healthcare and health services research. 

Finally, to best address ongoing debate in the field of SuMDs reprocessing, as well as adequately describing reprocessing oversight and processes, researchers should ensure that reprocessing safety and effectiveness studies are adequately powered to detect effects for primary and rare event outcomes e.g., major complications, which was lacking in several studies included in this review. Additionally, moving from observational to randomised controlled trials and adhering to relevant study design reporting standards would improve our confidence in the safety outcomes reported. When the proposed primary research is undertaken and reported as recommended, future systematic reviews on this topic could examine relationships between “reprocessing oversight” and safety, cost-effectiveness and environmental impacts. 

### Strengths and limitations

The strengths of this review are its broad focus and the rigorous methods employed. We attempt to consider the alignment of reprocessing with quality assurance standards in order to help contextualise similarities and differences in the findings between studies of similar risk SuMDs [36]. By using a modern definition of reprocessing to determine study eligibility for inclusion, we were able to eliminate risks of including studies of similar related practices (e.g. sterilisation only). By distinguishing between the different “levels” of reprocessing oversight across studies, there was a potential to explore trade-offs between reprocessing safety and cost savings outcomes by reprocessing oversight. This distinction was useful as reprocessing regulation often requires outsourcing of reprocessing from hospital sterilisation departments to third-party reprocessors [1], [2]. Conversely, it is possible that certain eligible items were excluded if they did not define “reprocessing” or report on the reprocessing procedures. Failure to report this information could add confusion to this topic and authors are encouraged to include these details in their studies. 

To ensure adequate clinical knowledge of individual SuMDs, advice was sought from the Health Products Regulatory Authority (HPRA), Ireland’s regulatory body for health products, including medical devices. 

Although standardising costs data to a single currency and for the current year to adjust for inflation is common in systematic reviews of economic studies [14], we felt that doing so would not result in comparable costs in this review due to the quality of the cost studies, the outcomes identified, the likely advances in technology, and regional differences in costs. Instead, the broader trend of the presence or absence of cost savings in individual studies comparing reused and once-used SuMDs was reported. 

## Conclusion

Insufficient quality evidence to establish the safety, cost-effectiveness and environmental impacts of reprocessing risk class I and risk class II SuMDs persists. Reprocessing results in cost savings and reduced global warming impacts but marginal savings diminish with subsequent reprocessing cycles. The volume and type of available evidence differs by device type. There is a need for explicit monitoring of the safety of risk class I and risk class IIa SuMD reprocessing where the practice is permitted under legislation. Reprocessing costs should be estimated using appropriate methodologies, and research is needed to enable life cycle assessment study designs to go through all critical stages of a systematic review to best utilise them to inform decision-making. 

## Notes

### Authors’ ORCIDs

- McGrath N: 0000-0002-7716-7277
- Waldron C: 0000-0002-0408-8543
- Farragher A: 0009-0007-6338-2447
- Teahan A: 0000-0002-4734-1457
- Keshtkar A: 0000-0001-5249-3589
- Tessarolo F: 0000-0003-4022-5602
- Polisena J: 0000-0002-1560-3651

### Source(s) of support

This work was funded by the Health Research Board Evidence Centre, which is funded by the Department of Ireland, Ireland.

### Role of the funder/sponsor

The funder had no role in the design and conduct of the study; collection, management, analysis, and interpretation of the data; preparation, review, or approval of the manuscript; and decision to submit the manuscript for publication.

### Disclaimer 

Any views expressed in this report are those of the authors and not necessarily those of Ireland’s Minister for Health, Ireland’s Department of Health, the Health Research Board, the Bruno Kessler Foundation or the International Federation for Medical and Biological Engineering. The review authors have no competing interests to declare. 

### Acknowledgements

We extend our thanks to our colleagues at the Health Products Regulatory Authority, James McCarthy, Patrick Murphy, Dhanashree Gokhale, and Jennifer Roche, to Dr Karen McNamara at the Department of Health and to our Health Information and Quality Authority colleagues Dr Kieran Walsh and, formerly, Dr Paul Carty for their conceptual input during the design phase of this review. Several of our colleagues within the Health Research Board Evidence Centre also provided invaluable assistance with individual components of the review; specifically, Jean Long contributed to the review conception and early draft review and Dr Annette Burns who contributed to the GRADE assessment and data presentation. Finally, we thank peer reviewers who provided considered and valuable feedback on the full report, of which the present article forms a component of; Dr Lisa Leung, MRCP and Dr Mark Gallagher, MD FRCPI

### Competing interests

The authors declare that they have no competing interests.

## Supplementary Material

Search strategies

Review outcome selection

Quality assessment

Meta-analysis feasibility assessment

Grading of recommendations, assessment, development and evaluations

## Figures and Tables

**Table 1:**
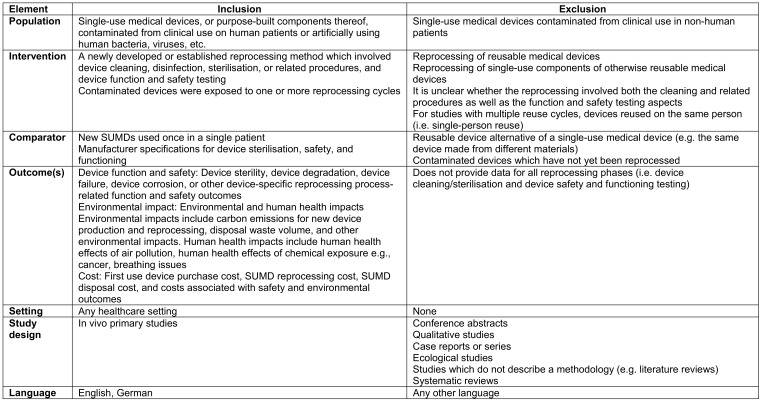
Systematic review eligibility criteria

**Table 2:**
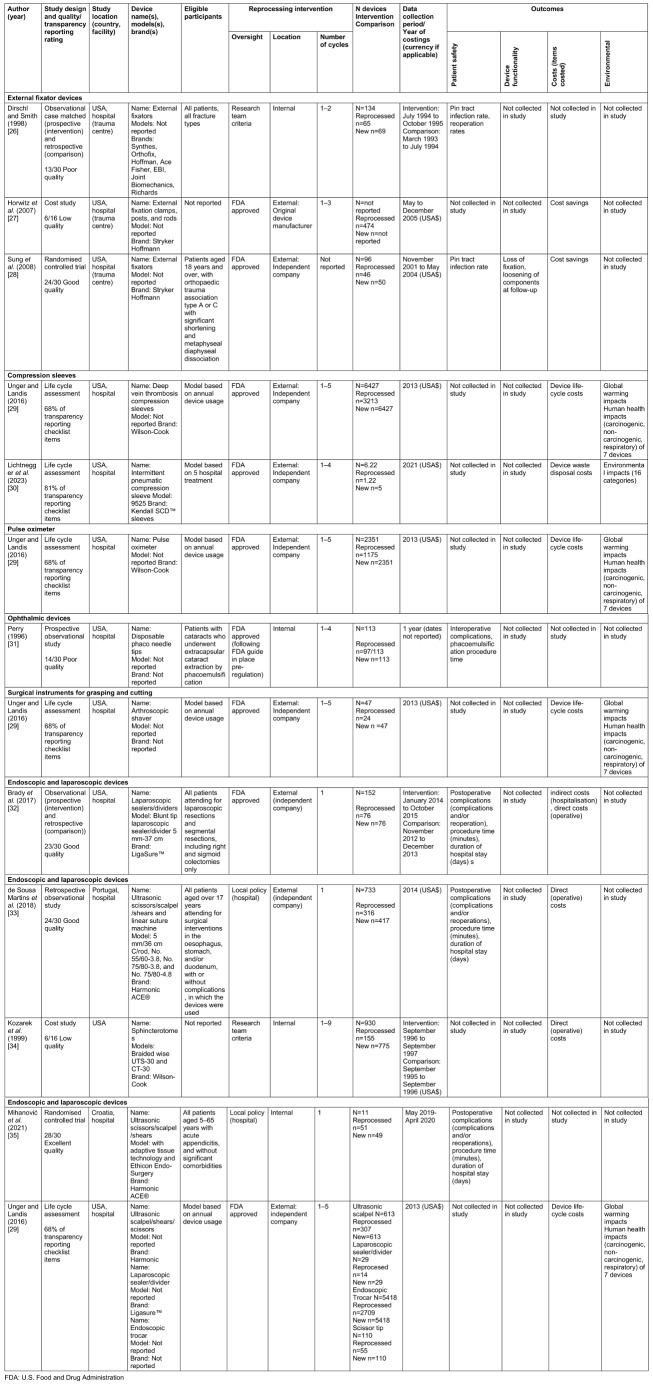
Characteristics of included studies

**Table 3:**
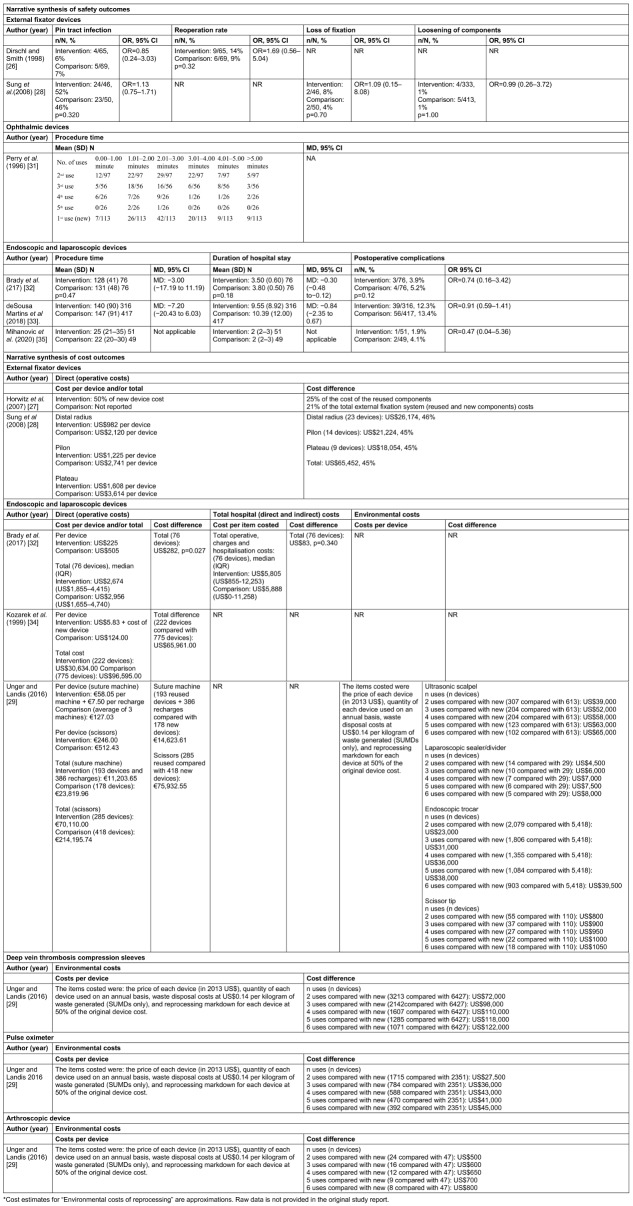
Narrative synthesis of safety and cost outcome data

**Table 4:**
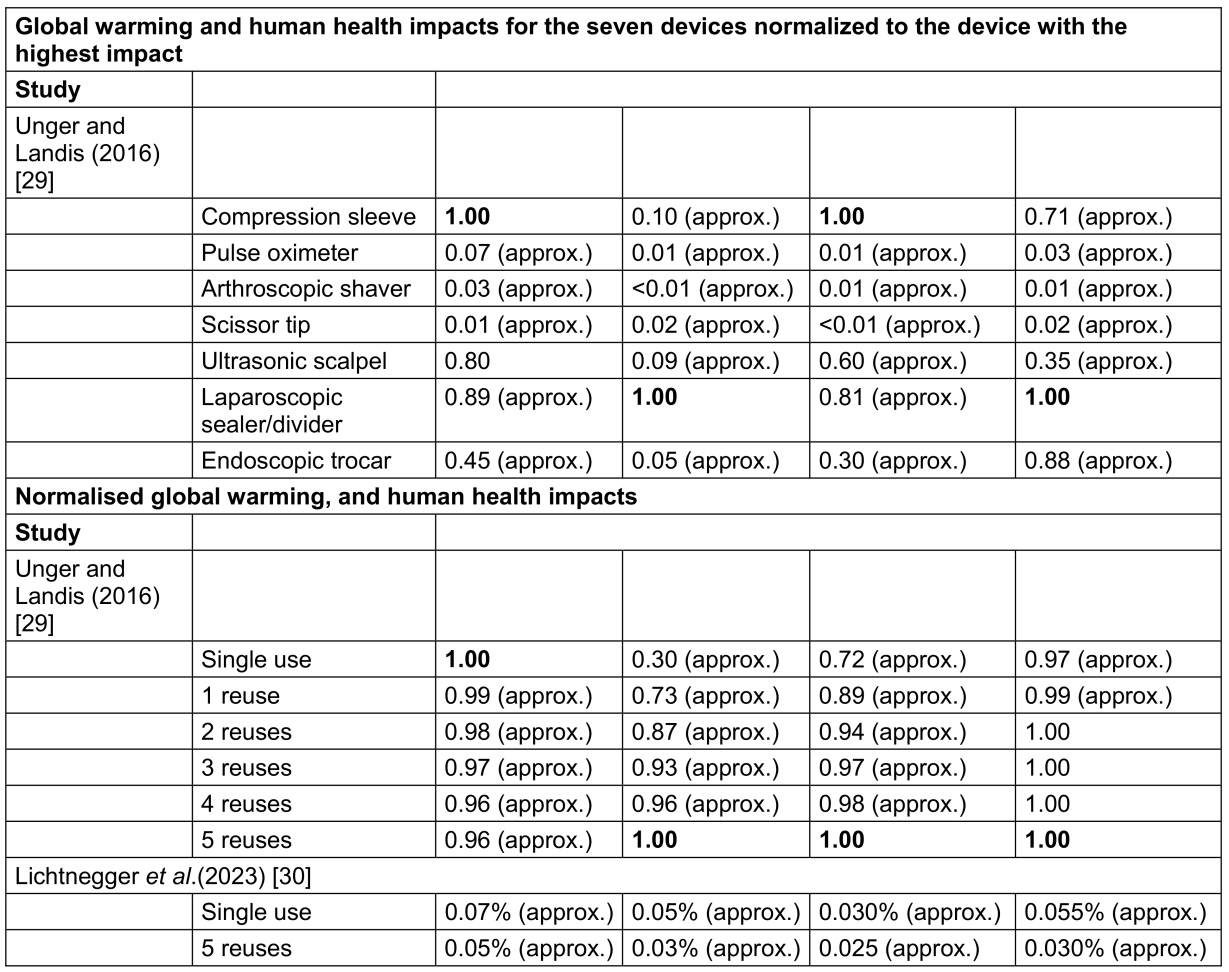
Narrative synthesis of environmental impact outcomes

**Table 5:**
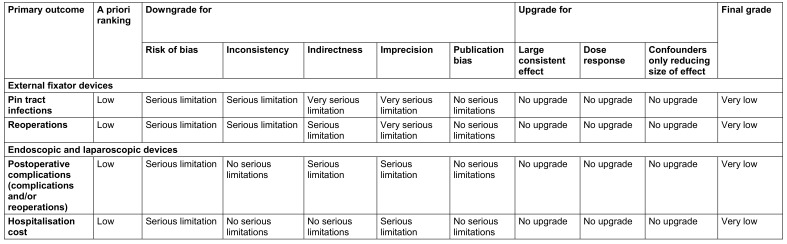
GRADE rating for primary outcomes
