# Supplementary material for: Safety, cost and environmental impact of reprocessing low and moderate risk single-use medical devices: a systematic review
Source: GMS Hyg Infect Control. 2026 Jan 26;21:Doc08. doi: 10.3205/dgkh000617 (PMC12921675; doi:10.3205/dgkh000617)
Supplement: Search strategies [file HIC-21-08-s-001.pdf]

Attachment 1

Search strategies

Table S1 Overview of database results

Database	Date of search	No. of results
**Ovid MEDLINE**	25 July 2022	3,617
**Embase***	25 July 2022	2,079
**Cochrane Trial Register**	25 July 2022	291
**Cochrane Library (John Wiley & Sons Inc.)**	25 July 2022	3
**Dimensions**	25 July 2022	304
**Total before deduplication**		6,294
**Total after deduplication**		5,041
**Total retained for analysis after screening**	Health setting	18
**Total retained for analysis after screening**	Laboratory setting	33
**Total added from reference chasing**		1

**primary review database*

Embase

Table S2 Embase search strategy 25 July 2022

Query number (#)	Search terms	Results
#46	#45 AND ('article'/it OR 'article in press'/it OR 'chapter'/it OR 'conference abstract'/it OR 'conference paper'/it OR 'preprint'/it OR 'review'/it OR 'short survey'/it)	2,079
#45	#30 AND #34 AND [1994-2022]/py	2,171
#44	#30 AND #34	2,269
#43	#35 NOT #42	2,268
#42	'single-use versus reusable medical devices in spinal fusion surgery':ti	1
#41	#35 NOT #40	2,268
#40	'a cost-effectiveness analysis of exalt model d single-use duodenoscope versus':ti	1
#39	#35 NOT #38	2,268
#38	'economic analysis of reprocessing single-use medical devices':ti	1
#37	#35 NOT #36	2,268

Query number (#)	Search terms	Results
#36	'systematic review of reusable versus disposable laparoscopic instruments':ti	1
#35	#30 AND #34	2,269
#34	#31 OR #32 OR #33	11,845,373
#33	#21 OR #22 OR #23 OR #24 OR #25 OR #26 OR #27 OR #28 OR #29	9,248,690
#32	#14 OR #15 OR #16 OR #17 OR #18 OR #19 OR #20	2,212,471
#31	#5 OR #6 OR #7 OR #8 OR #9 OR #10 OR #11 OR #12 OR #13	1,396,789
#30	#1 OR #2 OR #3 OR #4	2,947
#29	(equipment NEAR/5 (safety OR risk OR infection OR adverse OR malfunction)):ab,ti,kw	4,318
#28	(instrument NEAR/5 (safety OR risk OR infection OR adverse OR malfunction)):ab,ti,kw	2,472
#27	(device* NEAR/5 (safety OR risk OR infection OR adverse OR malfunction)):ab,ti,kw	22,199
#26	'instrument sterilization'/exp OR 'disinfection'/exp OR disinfect*:ab,ti,kw OR sterilz*:ab,ti,kw OR sterilis*:ab,ti,kw OR quarantin*:ab,ti,kw	89,657
#25	'infection'/exp OR 'hygiene'/exp OR 'cross infection'/exp	4,264,422
#24	'risk'/exp	2,850,294
#23	'adverse event'/exp	967,425
#22	'safety procedure'/exp	64,078
#21	'safety'/exp OR safety:ab,ti,kw OR incidence:ab,ti,kw OR mortality:ab,ti,kw	3,430,453
#20	'environmental aspects and related phenomena'/exp	2,075,373
#19	('waste' NEAR/5 (dispos* OR management OR cost OR impact)):ab,ti,kw	23,057
#18	(environment* NEAR/5 (dispos* OR management OR cost OR impact)):ab,ti,kw	50,468
#17	(waste OR environment*)	25,727
#16	'environmental sustainability'/exp OR 'environmental impact'/exp	95,197

Query number (#)	Search terms	Results
#15	'waste management'/exp	213,588
#14	'waste disposal'/exp	19,39
#13	(cost NEAR/5 (benefit* OR effectiv* OR comparat* OR analy*)):ab,ti,kw	278,703
#12	'cost benefit':ab,ti,kw OR 'cost-benefit':ab,ti,kw OR 'cost analysis':ab,ti,kw OR 'cost-analysis':ab,ti,kw OR 'cost compar*':ab,ti,kw OR 'cost implication*':ab,ti,kw OR 'cost effectiv*' OR 'cost-effectiv*':ab,ti,kw OR cost:ab,ti,kw	747,969
#11	'cost minimization analysis'/exp	3,797
#10	'hospital cost'/exp	43,215
#9	'health economics'/exp	987,523
#8	'health care financing'/exp	13,727
#7	'health care cost'/exp	323,080
#6	'cost control'/exp	73,496
#5	'cost benefit analysis'/exp	90,954
#4	((sud OR sumd) NEAR/20 (instrument* OR device* OR equipment)):ab,ti,kw	146
#3	('certified reprocessing' NEAR/20 (instrument* OR device* OR equipment)):ab,ti,kw	2
#2	((reuse OR 're-use' OR 're use' OR reprocess* OR remanufact*) NEAR/20 (instrument* OR device* OR equipment)):ab,ti,kw	1,807
#1	('single use' NEXT/10 (instrument* OR device* OR equipment)):ab,ti,kw	1,276

MEDLINE

Table S3 MEDLINE search strategy 25 July 2022

Query number (#)	Search terms	Results
#1	((reuse or re-use or reusing or reprocess* or remanufact* or resterili#e*) adj10 (instrument* or device* or equipment)).mp.	3,908
#2	(certified reproc* or sud or SUMD or (single adj use* adj10 (instrument* or device* or equipment))).mp.	7,633
#3	or/1-2	11,259
#4	exp Equipment Reuse/	3,158
#5	exp "Equipment and Supplies"/	1,610,810
#6	((medical or surgical) and (device* or instrument* or equipment)).mp.	293,141
#7	(single adj use* adj10 (instrument* or device* or equipment)).mp.	966
#8	exp Surgical Instruments/	25,809
#9	exp Surgical Equipment/	286,785
#10	exp disposable equipment/	5,265
#11	exp "Equipment and Supplies, Hospital"/	27,237
#12	or/4-11	1,775,417
#13	exp Cost-Benefit Analysis/	90,518
#14	exp "Cost Control"/	34,037
#15	health care cost effectiveness.mp.	15
#16	exp Healthcare Financing/	1,202
#17	exp "Health Care Economics and Organizations"/	1,642,271
#18	exp Hospital Costs/	11,865
#19	" cost minimi*ation analysis".mp.	808
#20	('cost benefit' or 'cost-benefit' or 'cost analysis' or 'cost-analysis' or 'cost compar*' or 'cost implication*' or 'cost-effectiv*' or cost).mp.	615,324
#21	(cost adj5 (benefit* or effectiv* or comparat* or analy*)).mp.	280,595
#22	or/13-21	2,004,556
#23	exp Carbon Footprint/	903
#24	(waste adj5 (dispos* or management or cost or impact)).mp.	56,496
#25	(environment* adj5 (dispos* or management or cost or impact)).mp.	42,189

Query number (#)	Search terms	Results
#26	((environment* adj5 sustainab*) or carbon footprint or recycl*).mp.	72,242
#27	or/23-26	158,865
#28	exp Safety Management/ or Safety Management.mp.	22,460
#29	exp Patient Safety/	24,697
#30	exp Equipment Safety/	10,437
#31	((device or equipment) adj5 (defect* or failure* or misuse or malfunction*)).mp.	66,479
#32	((contaminat* or complicat* or erosion) adj5 (instrument* or device* or equipment)).mp.	29,419
#33	(safety or incidence or mortality).mp.	2,718,406
#34	(Adverse event or Adverse Event Outcome).mp.	34,945
#35	exp "Risk Evaluation and Mitigation"/	64
#36	exp Risk/	1,350,136
#37	(Infection Control or Infection, Healthcare Associated).mp.	47,963
#38	exp Hygiene/ or exp Cross Infection/	104,935
#39	exp instrument sterilization/ or exp disinfection/ or (disinfect* or sterilz* or sterilis* or quarantin*).mp.	70,970
#40	exp Sterilization/ or (sterili* adj5 (instrument* or device* or equipment)).mp.	34,448
#41	or/28-40	3,867,074
#42	(substance adj5 disorder*).mp.	117,748
#43	22 or 27 or 41	5,675,733
#44	3 and 43	5,423
#45	limit 44 to yr="1994 -Current"	5,203
#46	limit 45 to (comment or editorial or letter or newspaper article)	276
#47	45 not 46	4,927
#48	"substance use".ti,ab,kw.	45,398
#49	47 not 48	3,617

Database(s): **Ovid MEDLINE and Epub Ahead of Print, In-Process, In-Data-Review and Other Non-Indexed Citations, Daily and Versions** 1946 to July 25, 2022

Dimensions database

Table S4 Dimensions database search strategy 25 July 2022

Search term	Results
("reprocessing device"~5) OR ("reuse device" ~5) OR ("resterili?e device"~5)<br>Filter: Title and Abstract search	304

Cochrane Library and Register

Table S5 Cochrane Library (John Wiley & Sons Inc.) search strategy 25 July 2022

Query number (#)	Search terms	Results
#1	(reuse OR re-use or reprocess* or remanufact*) and ((SUD or SUMD* or single-use or "single use") NEAR/5 (device* or instrument* or equipment))	14
#2	MeSH descriptor: [Equipment and Supplies] explode all trees	53,231
#3	MeSH descriptor: [Equipment Reuse] this term only	101
#4	MeSH descriptor: [Equipment and Supplies] this term only	186
#5	[mh "Equipment and Supplies"[mj]]	8,347
#6	#1 or #4 or #3	294

Grey literature table

General scoping searches were carried out on the Google.com search engine to gain an initial idea of terminology and likely key terms. Reviewing literature in the area (retrieved from the Epistemonikos and Cochrane databases) also helped build up our search vocabulary. Initial search terms used included combinations of ‘single-use device’, ‘SUD’, ‘SUMD’, and ‘single-use medical device’, together with language around reprocessing and reuse. Further searches were carried out using the websites of relevant bodies (see *Table S6*).

Table S6 Websites included in supplementary grey literature search

Organisation	Website
Bundes Gesundheit Ministerium (Federal Ministry of Health, Germany)	https://www.bundesgesundheitsministerium.de/en/ministry/the-federal-ministry-of-health.html
Center for the Evaluation of Value and Risk in Health (Cost-Effectiveness Analysis (CEA) Registry, Tufts University, Boston, Massachusetts, United States of America (USA))	https://cevr.tuftsmedicalcenter.org/databases/cea-registry
Centre for Reviews and Dissemination (CRD, University of York, United Kingdom (UK))	https://www.york.ac.uk/crd/
Competent Authorities for Medical Devices (CAMD)	https://www.camd-europe.eu/
Department of Health, Ireland	https://www.gov.ie/en/organisation/department-of-health/#
European Commission	https://ec.europa.eu/info/index_en
European Union (EU) Law (Europa)	https://european-union.europa.eu/institutions-law-budget/law_en
European database on medical devices (EUDAMED)	https://ec.europa.eu/tools/eudamed/#/screen/home
Google	https://www.google.ie/
International Health Technology Assessment Database	https://www.inahta.org/
Health Systems Evidence	https://www.healthsystemsevidence.org/
Lenus	https://www.lenus.ie/
Livivo	https://www.livivo.de
MedTech Europe (trade association)	https://www.medtecheurope.org/
OpenGrey repository	http://www.opengrey.eu
PROSPERO registry	https://www.crd.york.ac.uk/prospero/
World Health Organization	https://www.who.int/
