# Supplementary material for: Safety, cost and environmental impact of reprocessing low and moderate risk single-use medical devices: a systematic review
Source: GMS Hyg Infect Control. 2026 Jan 26;21:Doc08. doi: 10.3205/dgkh000617 (PMC12921675; doi:10.3205/dgkh000617)
Supplement: Review outcome selection [file HIC-21-08-s-002.pdf]

Attachment 2

Review outcome selection

Table S*1* and Table S*3* show the full spectrum of outcomes collected in eligible studies. Items in red font denote studies or individual outcomes excluded at this stage. We excluded studies which did not report on patient safety or only provided patient or device safety outcomes via subjective measurement methods (e.g. surgeon opinion). For many outcomes, we provided a note (highlighted in yellow) indicating a possible strategy of combining and grouping outcomes. This is not indicative of finalised outcome grouping decisions. One study was excluded during this outcome selection process.

Table S1 Safety outcome selection and preliminary groupings

**Author**	**Safety outcome name**	**Outcome type**	**Definition and note on preliminary grouping**
**Endoscopic and laparoscopic devices**			
Brady *et al.* (2017) [1]	Estimated blood loss	Patient safety (direct)	As name
	Additional interventions required for vascular pedicle ligation	Patient safety (direct)	Additional interventions were defined as any application of additional monopolar or bipolar energy after the initial ligation, the application of clips, or a stapling device. COMPLICATIONS (during procedure)
	Efficiency: by comparing operative time between groups	Patient safety (indirect)	As name (procedure time)
	Length of hospital stay	Patient safety (indirect)	As name
	Reoperations	Patient safety (direct)	As name COMPLICATIONS
de Sousa *et al.* (2018) [2]	3. Clinical efficiency: duration of surgical intervention	Patient safety (indirect)	As name (procedure time)
	6. Postoperative infection incidence	Patient safety (direct)	Postoperative infection incidence up to 30 days after surgery COMPLICATIONS
	7. Antibiotic consumption	Patient safety (indirect)	Antibiotic consumption (using the daily dose defined) up to 30 days after surgery COMPLICATIONS
	5. Reoperations	Patient safety (direct)	No patients requiring reoperation up to 30 days after surgery COMPLICATIONS

Author	Safety outcome name	Outcome type	Definition and note on preliminary grouping
	4. Length of hospital stay	Patient safety (indirect)	Up to 30 days after surgery
	8. In-hospital mortality	Patient safety (direct)	Up to 30 days after surgery COMPLICATION
	9. Re-hospitalisation rate	Patient safety (direct)	Up to 30 days after surgery COMPLICATION
Mihanović *et al.* (2021) [3]	10. Speed of transection of the appendiceal base	Device function (direct)	As name
	5. Complications (intraoperative, postoperative, reoperations)	Patient safety (direct)	As name COMPLICATIONS – COMBINE ALL FOR INTRAOPERATIVE, POSTOPERATIVE, REOPERATIONS
	Subjective assessment of the surgeon about the instrument	Device function (direct)	Hemostasis, coagulation efficiency, cutting efficiency, force applied for dissection, error messages/disturbing notes (subjective assessment)
	Duration of surgery	Patient safety (indirect)	As name (procedure time)
	Duration of hospital stay	Patient safety (indirect)	As name (until discharge)
**External fixator devices**			
Dirschl and Smith (1998) [4]	Pin tract infection rate	Patient safety (direct)	As name
	Reoperation after external fixation	Patient safety (direct)	As name
	Device failure rate	Device failure (direct)	As name (unclear – no further details)
Sung *et al.* (2008) [5]	Pin tract infections	Patient safety (direct)	Any site of purulence, erythema, or drainage. If any pin site in a patient showed these signs, it was considered a positive finding – as Dirschl and Smith
	Loosening during follow-up	Device function (direct)	Loosening was determined clinically by gross motion at the pin site.
	Loss of fixation	Device failure (direct)	Loss of fixation (as determined by the attending surgeon) was defined by a change in the radiographic alignment of the fracture (greater than 5 degrees or any shortening were the criteria so as to account for varying radiographic views)
**Ophthalmic devices**			
Perry (1996) [6]	Number of phacoemulsification (phaco) tip uses	Device function (indirect)	Never used more than five times. Assessed before use under the operative microscope and for integrity.
	Phacoemulsification time	Device function (direct)	As name

Author	Safety outcome name	Outcome type	Definition and note on preliminary grouping
	Nuclear sclerosis	Patient safety (direct)	Of the cataract with each use
	Problems related to needle tip	Patient safety (direct)	Intraoperative problems during the procedure
**Diathermy devices**			
Loftus (2015) [7]	Reported defects	Device function (direct)	Any time a member of the surgical team (surgeon, scrub technician, first assistant, or circulating nurse) determined that the bipolar and ultrasound diathermy device was not functioning in a manner consistent with the devices' intended purpose (subjective measure).

We also reviewed studies contributing cost data to determine the eligibility of available outcome data for this review. Table S2 reports the criteria used by Health Research Board (HRB) reviewers (ÁT and NMG) to determine the eligibility of cost outcomes, and our final decisions on same.

Table S3 Selection of cost outcomes

Author (Year)	Transparent methods	Actual costs used	Costing source	Other comments	Findings	HRB inclusion decision
**Endoscopic and laparoscopic devices**						
Brady *et al.* (2017) [1]	No – lack of information on costing sources	Yes<br><br>Operative (device cost; time)<br><br>Postoperative (length of stay; reoperation)	Hospital Chief Financial Officer	In 19.7% of cases, surgeon was dissatisfied with reprocessed device.	No significant increase in hospital profit margin	Keep
de Sousa *et al.* (2018) [2]	Yes	Yes<br><br>Device cost only	Actual cost of reprocessed versus new device	Postoperative factors (surgery duration, hospital stay, re-hospitalisation) all insignificant between reprocessed and new device groups	A total of 193 linear suturing machines (GIA Covidien™) were reprocessed, saving €14,623.61.<br><br>Of the ultrasonic scalpel/shears/scissors (Harmonic ACE®), 285 were reprocessed, corresponding to savings of €75,932.55.	Keep
**External fixator devices**						
Dirschl and Smith (1998) [4]	No	Yes<br><br>Pre-operative: Nurse training cost<br><br>Operative: Device cost	Not reported	None	The overall mean hospital charge for an external fixation device decreased 32% as a result of the reuse programme, from US$4,067 (US dollars) before reuse (range: US$2,009–10,002) to US$2,791 after reuse (range: US$1,106–10,415).	Reject – lack of clarity on methods
Sung *et al.* (2008) [5]	No	Yes – device cost only	Hospital purchasing department	It would take 1,600 patients per arm to truly demonstrate equivalence with 80% power based on our pilot study.	Actual savings of US$65,452	Keep
