# Supplementary material for: Safety, cost and environmental impact of reprocessing low and moderate risk single-use medical devices: a systematic review
Source: GMS Hyg Infect Control. 2026 Jan 26;21:Doc08. doi: 10.3205/dgkh000617 (PMC12921675; doi:10.3205/dgkh000617)
Supplement: Quality assessment [file HIC-21-08-s-003.pdf]

Attachment 3

Quality assessment

Details of adaptations to the Downs and Black checklist

The original Downs and Black checklist consists of 27 items across the following methodological components: reporting, external validity, internal validity (bias and confounding), and power. Twenty-six items were rated either as yes (1) or no/unable to determine (0), and one item was rated on a 3-point scale (yes=2, partial=1, and no=0). The checklist has been ranked in the top six quality assessment tools suitable for use in systematic reviews [8] and has adequate internal consistency, test–retest reliability, inter-rater reliability and criterion validity.

We added the question "Was an attempt made to blind SUD user(s) to the intervention they delivered?", rated either as yes (1) or no/unable to determine (0), to capture performance bias of those implementing SUD reprocessing. We also adapted the scoring for the question "Did the study have sufficient power to detect a clinically important effect where the probability value for a difference being due to chance is less than 5%?", rating as yes (2) where the study was powered to detect a difference for at least one-half of the outcomes, including the primary outcome; partially (1) where the study was powered to detect a difference for the primary outcome only; and no/unable to determine (0) where the study was not powered to detect a difference for any outcome, or we could not tell whether power calculations were undertaken. These adaptations resulted in an overall total possible score of 30. We adapted our quality ratings to allow for the score changes as follows: excellent (27–30), good (21–26), fair (16–20), and poor (≤15); these ratings are in line with previously suggested categories [9].

Details of adaptations to the Consensus Health Economic Criteria list (CHEC-list)

The CHEC-list contains 19 questions on different aspects of economic evaluations: for example, study design; time horizon; study perspective; type of costs and effectiveness measures that are included; the way these costs are measured and valued; incremental analysis of costs and outcomes; discounting; sensitivity analyses; authors' conclusions; and generalisability of study results. Each question can be answered 'yes' or 'no'. If the answer is 'yes', this means that the study either adequately performed the item of concern or reported the item in an appropriate way [10].

The economic studies identified in this systematic review were classified as cost studies rather than full economic evaluations, as study authors used a simple cost-calculator approach where they made various assumptions about the inputs to investigate whether these assumptions affected the overall estimates. Since there are currently no quality appraisal tools specifically designed for these types of studies, adaptations to the CHEC-list were necessary in order to facilitate quality appraisal. Adaptations were made in consultation with two health economists (ÁT and PC) and informed by the Jacobs *et al.* review on this topic which adopted a similar approach to quality appraisal [11]. Specifically, we adapted Question 5 of the CHEC-list to read "Is the chosen time horizon/**duration of study observation period** appropriate to include relevant costs and consequences?" in order to reflect that the time horizon in studies included in this review was derived from the observation period. We removed the questions "Were all outcomes measured appropriately?", "Were all outcomes valued appropriately?", and "Are all future costs and outcomes discounted appropriately?" in line with the Jacobs *et al.* review [11] and given the absence of discounting in these studies. This resulted in a total possible score of 16, with quality ratings of high (>75% of items receiving a score of 1), moderate (between >50% and ≤75% of items receiving a score of 1), and low (≤50% of items receiving a score of 1), which is in keeping with previous research [12–14].

Results of quality appraisals for trials and comparative observational and cost studies

Table S9 Quality assessment ratings for trials and comparative observational studies

	External fixator devices		Endoscopic and laparoscopic devices			Opthalmic devices
Item	Dirschl and Smith (1998) [4]	Sung *et al.* (2008) [5]	Brady *et al.* (2017) [1]	de Sousa *et al.* (2018) [2]	Mihanović *et al.* (2021) [3]	Perry (1996) [6]
1. Aim/objectives stated	1	1	1	0	1	0
2. Main outcomes stated before results	0	1	1	1	1	1
3. Observation characteristics clearly described	0	1	1	1	1	0
4. Interventions clearly described	1	1	1	1	1	1
5. Distributions of confounders clearly described	0	2	1	1	2	0
6. Main findings clearly described	0	1	1	1	1	1
7. Estimates of random variability	1	1	1	1	1	1
8. Adverse events reported	1	1	1	1	1	0
9. Patients lost to follow-up described	1	0	1	1	0	0
10. Exact probability values reported	0	1	1	1	1	1
11. Subjects representative of the entire population	1	1	1	1	0	1
12. Subjects representative of population recruited	1	0	1	1	1	1
13. Treatment representative of what the majority of patients receive	1	1	1	1	1	1
14. Attempt made to blind subjects	0	1	1	1	1	0
15. Attempt made to blind single-use device user(s)	0	0	0	0	1	0
16. Attempt made to blind those measuring outcomes	1	0	1	1	1	0
17. ‘Data dredging’ made clear	1	1	1	1	1	1
18. Analyses adjusted for follow-up	0	0	1	1	0	1
19. Statistical tests appropriate	0	1	1	1	1	0
20. Compliance reliable	1	1	0	1	1	1
21. Outcome measures accurate	0	1	1	1	1	0
22. Patients recruited from the same population	1	1	1	1	1	1
23. Subject recruited over the same period	0	1	1	1	1	1
24. Randomised	0	1	0	0	1	0
25. Assignment concealed	0	1	0	0	1	0
26. Adjustment for confounding	0	1	1	1	1	0
27. Losses to follow-up considered	1	1	1	1	1	1
28. Power to detect effect	0	0	0	0	1	0
**Total score out of 30**	**13**	**24**	**23**	**24**	**28**	**14**

Table S1 Quality assessment ratings for cost studies

**Cost quality assessment**	**External fixator devices**	**Endoscopic and laparoscopic devices**
	Horwitz et al. (2007) [15]	Kozarek et al. (1999) [16]
1. Is the study population clearly described?	0	0
2. Are competing alternatives clearly described?	1	1
3. Is a well-defined research question posed in answerable form?	0	1
4. Is the economic study design appropriate to the stated objective?	0	0
5. Is the chosen time horizon/duration of study observation period appropriate to include relevant costs and consequences?	0	0
6. Is the actual perspective chosen appropriate?	1	1
7. Are all important and relevant costs for each alternative identified?	0	0
8. Are all costs measured appropriately in physical units?	1	1
9. Are costs valued appropriately?	1	0
10. Are all important and relevant safety outcomes for each alternative identified?	0	0
11. Is an incremental analysis of costs and outcomes of alternatives performed?	1	0
12. Are all important variables, whose values are uncertain, appropriately subjected to sensitivity analysis?	0	0
13. Do the conclusions follow from the data reported?	0	1
14. Does the study discuss the generalisability of the results to other settings and patient/client groups?	0	1
15. Does the article indicate that there is no potential conflict of interest of study researcher(s) and funder(s)?	1	0
16. Are ethical and distributional issues discussed appropriately?	0	0
**Total score out of 16**	**6**	**6**

Table S2 Transparency reporting for life cycle assessment studies

Checklist item	Score (0, 0.5, 1.0)
	Unger and Landis (2016) [17]
Does the study specify its goal?	0.5
What kind of product(s) is examined?	0.5
Is the functional unit described?	1.0
What scope is used in this study?	1.0
Does the study describe the process modules with qualitative and quantitative data?	1.0
Does the study specify excluded processes?	1.0
Does the study specify data quality requirements?	0.0
Which region is used as the reference region?	1.0
What year is used as the reference year?	1.0
Does the study specify the data sources for primary data?	1.0
Does the study specify the data sources for secondary data?	1.0
What allocation method(s) was (were) used?	1.0
Was the final life cycle inventory model made available?	0.0
What midpoint impact categories are used?	0.5
Does the study report the used impact category or classification and characterisation?	1.0
Does the study report the total results of the examined products?	1.0
Does the study report the results for each life cycle phase?	0.0
Does the study report an uncertainty analysis?	0.0
Does the study report a sensitivity analysis?	1.0
Does the study discuss its limitations?	0.0
Does the study state a funding source and its role?	0.5
Does the study state that an external critical review was performed?	1.0
Total score out of 22	15.0
