# Supplementary material for: Safety, cost and environmental impact of reprocessing low and moderate risk single-use medical devices: a systematic review
Source: GMS Hyg Infect Control. 2026 Jan 26;21:Doc08. doi: 10.3205/dgkh000617 (PMC12921675; doi:10.3205/dgkh000617)
Supplement: Meta-analysis feasibility assessment [file HIC-21-08-s-004.pdf]

Attachment 4

Meta-analysis feasibility assessment

Table S1 Feasibility assessment for meta-analysis of individual outcomes

Outcome	Number of studies (>3)	Low assessment of quality or of risk of bias (bias in blinding, randomisation, missing outcome data, outcome assessment)	Population, intervention, comparator, outcome(s), time frame, and study design (PICOTS) assessment (clinical and methodological diversity)					Meta-analysis feasibility decision
			Population (eligibility, key demographics)	Intervention and comparator	Outcome (definition and means of reporting)	Study design	Time frame	
External fixator devices								
Pin tract infections	2 studies: Dirschl and Smith (1998) [4] and Sung *et al.* (2008) [5]	Different<br><br>Dirschl and Smith (1998): 13/30<br><br>Sung *et al.* (2008): 24/30	Similar eligibility<br><br>Sung *et al.* (2008): Aged 18 years and over, could consent, functioned independently, lived locally, sustained a fracture of the humerus, distal radius, wrist, femur, tibia, or ankle for which external fixation was the chosen initial treatment<br><br>Dirschl and Smith (1998): All patients with external fixation devices applied at the study centre within the study period	Similar device(s)<br><br>Both: Stryker Hoffman (+6 more in Dirschl and Smith (1998))<br><br>Different locations<br><br>Dirschl and Smith (1998): Internal<br><br>Sung *et al.* (2008): External	Definition similarity unclear<br><br>Dirschl and Smith (1998): Not reported<br><br>Sung *et al.* (2008): Any site of purulence, erythema, or drainage<br><br>Similar measurement<br><br>Dirschl and Smith (1998): N, derive % (not reported by reprocessing cycle)	Different<br><br>Sung *et al.* (2008): Randomised controlled trial (RCT)<br><br>Dirschl and Smith (1998): Non-randomised controlled trial (NRCT)	Unclear similarity<br><br>Sung *et al.* (2008): 1–20 weeks<br><br>Dirschl and Smith (1998): Not reported	Does not meet criteria – too few studies

			Demographics similarity unclear<br><br>Age: Dirschl and Smith (1998): Not reported<br><br>Sung *et al.* (2008): 45 years<br><br>% female: Dirschl and Smith (1998): Not reported<br><br>Sung *et al.* (2008): 26%	Different number of reprocessing cycles<br><br>Dirschl and Smith (1998): 1–2<br><br>Sung *et al.* (2008): 1	Sung *et al.* (2008): N, %			
Device failure rate	2 studies: Dirschl and Smith (1998) [4] and Sung *et al.* (2008) [5]	Different<br><br>Dirschl and Smith (1998): 13/30<br><br>Sung *et al.* (2008): 24/30	Similar eligibility<br><br>Sung *et al.* (2008): Aged 18 years and over, could consent, functioned independently, lived locally, sustained a fracture of the humerus, distal radius, wrist, femur, tibia, or ankle for which external fixation was the chosen initial treatment<br><br>Dirschl and Smith (1998): All patients with external fixation devices applied at the study centre within the study period<br><br>Demographics similarity unclear	Similar device(s)<br><br>Both: Stryker Hoffman (+6 more in Dirschl and Smith (1998))<br><br>Different locations<br><br>Dirschl and Smith (1998): Internal<br><br>Sung *et al.* (2008): External<br><br>Different number of reprocessing cycles	Definition unclear:<br><br>Dirschl and Smith (1998): Mechanical or other failure<br><br>Sung *et al.* (2008): Loss of fixation and loosening during follow up<br><br>Similar measurement<br><br>Dirschl and Smith (1998): n, derive % (not reported by reprocessing cycle)	Different<br><br>Sung *et al.* (2008): RCT<br><br>Dirschl and Smith (1998): NRCT	Unclear time frame<br><br>Sung *et al.* (2008): 1–20 weeks<br><br>Dirschl and Smith (1998): Not reported	Does not meet criteria – too few studies

			Age: Dirschl and Smith (1998): Not reported<br><br>Sung *et al.* (2008): 45 years<br><br>% female: Dirschl and Smith (1998): Not reported<br><br>Sung *et al.* (2008): 26%	Dirschl and Smith (1998): 1–2<br><br>Sung *et al.* (2008): 1	Sung *et al.* (2008): n, %			
Reoperations	1 study: Dirschl and Smith (1998) [4]	N/A	N/A	N/A	N/A	N/A	N/A	Does not meet criteria – too few studies
Ophthalmic devices								
Needle tip issues	1 study: Perry (1996) [6]	N/A	N/A	N/A	N/A	N/A	N/A	Does not meet criteria – too few studies
Phacoemulsification time	1 study: Perry (1996) [6]	N/A	N/A	N/A	N/A	N/A	N/A	Does not meet criteria – too few studies
Endoscopic and laparoscopic devices								
Procedure time	3 studies:<br><br>Brady *et al.* (2017) [1]	Similar<br><br>Brady *et al.* (2017): 23/30	Similar eligibility<br><br>Brady *et al.* (2017): Patients attending for laparoscopic	Broadly similar devices/procedures<br><br>Brady *et al.* (2017): LigaSure Sealer/Divider 5 mm–37 cm for	Same definition	Different<br><br>Brady *et al.* (2017): NRCT	Similar<br><br>Brady *et al.* (2017):	Does not meet criteria – different measurements, non-normal

Mihanović *et al.* (2021) [3]	Mihanović *et al.* (2021): 28/30	resections of right and sigmoid colectomies	laparoscopic colorectal surgery	Different measurements (minutes)	Mihanović *et al.* (2021): RCT	Procedure duration	outcome distribution
de Sousa *et al.* (2018) [2]	de Sousa *et al.* (2018): 24/30	Mihanović *et al.* (2021): All patients with acute appendicitis<br><br>de Sousa *et al.* (2018): All surgical interventions using ultrasonic scalpel/shears/scissors (Harmonic ACE®) and the linear suture machine (GIA Covidien™) with cut and anastomosis (various regions of body)	Mihanović *et al.* (2021): Ultrasonic scalpel/shears/scissors for laparoscopic appendectomy<br><br>de Sousa *et al.* (2018): Ultrasonic s scalpel/shears/scissors (Harmonic ACE®) for digestive or thoracic surgery (intestines, stomach, oesophagus)	Brady *et al.* (2017): μ, standard deviation (SD) (unadjusted)<br><br>de Sousa *et al.* (2018): μ, SD (unadjusted)<br><br>Mihanović *et al.* (2021): (Adjusted) median, interquartile range (IQR)	de Sousa *et al.* (2018): NRCT	Mihanović *et al.* (2021): Procedure duration<br><br>de Sousa *et al.* (2018): Procedure duration	
		Different demographics	Different locations	de Sousa *et al.* (2018) report overall and by diagnosis-related group			
		Age: Brady *et al.* (2017): 66 years	Brady *et al.* (2017): External				
		Mihanović *et al.* (2021): 15 years	Mihanović *et al.* (2021): Internal				
		de Sousa *et al.* (2018): 57 years	de Sousa *et al.* (2018): External				
		% female: Brady *et al.* (2017): 50%					
		Mihanović *et al.* (2021): 20%	Same number of reprocessing cycles:				

			de Sousa *et al.* (2018): 60%<br><br>Health status: Brady *et al.* (2017): Body mass index (BMI) 30<br><br>Mihanović *et al.* (2021): BMI 20<br><br>de Sousa *et al.* (2018): Not reported	Brady *et al.* (2017): 1<br><br>Mihanović *et al.* (2021): 1<br><br>de Sousa *et al.* (2018): 1				
Duration of hospital stay	3 studies:<br><br>Brady *et al.* (2017) [1]<br><br>Mihanović *et al.* (2021) [3]<br><br>de Sousa *et al.* (2018) [2]	Similar<br><br>Brady *et al.* (2017): 23/30<br><br>Mihanović *et al.* (2021): 28/30<br><br>de Sousa *et al.* (2018): 24/30	Similar eligibility<br><br>Brady *et al.* (2017): Patients attending for laparoscopic resections of right and sigmoid colectomies<br><br>Mihanović *et al.* (2021): All patients with acute appendicitis<br><br>de Sousa *et al.* (2018): All surgical interventions performed in 2014 in which ultrasonic scalpel/shears/scissors (Harmonic ACE® -5 mm/36 cm C/rod) and the linear suture machine GIA Covidien™ with cut and anastomosis (No. 55/60-3.8, No. 75/80-3.8, and No. 75/80-	Broadly similar devices/procedures<br><br>Brady *et al.* (2017): LigaSure Sealer/Divider 5 mm–37 cm for laparoscopic colorectal surgery<br><br>Mihanović *et al.* (2021): Ultrasonic scalpel/shears/scissors for laparoscopic appendectomy<br><br>de Sousa *et al.* (2018): Ultrasonic scalpel/shears/scissors (Harmonic ACE®) for digestive or thoracic surgery (intestines, stomach, oesophagus)	Same definition<br><br>Different measurements (days)<br><br>Brady *et al.* (2017): μ, SD (unadjusted)<br><br>de Sousa *et al.* (2018): μ, SD (unadjusted)<br><br>Mihanović *et al.* (2021): Median, IQR	Different<br><br>Brady *et al.* (2017): NRCT<br><br>Mihanović *et al.* (2021): RCT<br><br>de Sousa *et al.* (2018): NRCT	Similar<br><br>Brady *et al.* (2017): Length of stay<br><br>Mihanović *et al.* (2021): Length of stay<br><br>de Sousa *et al.* (2018): Length of stay	Does not meet criteria – different measurements, non-normal outcome distribution

	4.8) were used (various regions of body)<br><br>Different demographics<br><br>Age: Brady *et al.* (2017): 66 years<br><br>Mihanović *et al.* (2021): 15 years<br><br>de Sousa *et al.* (2018): 57 years<br><br>% female: Brady *et al.* (2017): 50%<br><br>Mihanović *et al.* (2021): 20%<br><br>de Sousa *et al.* (2018): 60%<br><br>Health status:<br><br>Brady *et al.* (2017): BMI 30<br><br>Mihanović *et al.* (2021): BMI 20<br><br>de Sousa *et al.* (2018): Not reported	Different locations<br><br>Brady *et al.* (2017): External<br><br>Mihanović *et al.* (2021): Internal<br><br>de Sousa *et al.* (2018): External<br><br>Same number of reprocessing cycles:<br><br>Brady *et al.* (2017): 1<br><br>Mihanović *et al.* (2021): 1<br><br>de Sousa *et al.* (2018): 1

Complications (infections, additional interventions, reoperations)	3 studies:<br><br>Brady *et al.* (2017) [1]<br><br>Mihanović *et al.* (2021) [3]<br><br>de Sousa *et al.* (2018) [2]	Similar<br><br>Brady *et al.* (2017): 23/30<br><br>Mihanović *et al.* (2021): 28/30<br><br>de Sousa *et al.* (2018): 24/30	Similar eligibility<br><br>Brady *et al.* (2017): Patients attending for laparoscopic resections of right and sigmoid colectomies<br><br>Mihanović *et al.* (2021): All patients with acute appendicitis<br><br>de Sousa *et al.* (2018): All surgical interventions performed in 2014 in which ultrasonic scalpel/shears/scissors (Harmonic ACE® (5 mm/36 cm C/rod) and the linear suture machine GIA Covidien™ with cut and anastomosis (No. 55/60-3.8, No. 75/80-3.8, and No. 75/80-4.8) were used (various regions of body)<br><br>Different demographics<br><br>Age: Brady *et al.* (2017): 66 years<br><br>Mihanović *et al.* (2021): 15 years	Broadly similar devices/procedures<br><br>Brady *et al.* (2017): LigaSure Sealer/Divider 5 mm–37 cm for laparoscopic colorectal surgery<br><br>Mihanović *et al.* (2021): Ultrasonic scalpel/shears/scissors for laparoscopic appendectomy<br><br>de Sousa *et al.* (2018): Ultrasonic scalpel/shears/scissors (ACE®) for digestive or thoracic surgery (intestines, stomach, oesophagus)<br><br>Different locations<br><br>Brady *et al.* (2017): External<br><br>Mihanović *et al.* (2021): Internal<br><br>de Sousa *et al.* (2018): External	Different definitions<br><br>de Sousa *et al.* (2018): Infections, reoperations, re-hospitalisations<br><br>Mihanović *et al.* (2021): Complications (intraoperative, postoperative, reoperations)<br><br>Brady *et al.* (2017): Additional interventions required, reoperations<br><br>Similar reporting<br><br>de Sousa *et al.* (2018): %<br><br>Mihanović *et al.* (2021): n, %<br><br>Brady *et al.* (2017): n, %	Different<br><br>Brady *et al.* (2017): NRCT<br><br>Mihanović *et al.* (2021): RCT<br><br>de Sousa *et al.* (2018): NRCT	Different time periods<br><br>de Sousa *et al.* (2018): 30 days<br><br>Mihanović *et al.* (2021): 30 days<br><br>Brady *et al.* (2017): Surgery to discharge	Does not meet criteria for meta-analysis – different devices

de Sousa *et al.* (2018): 57 years<br><br>% female: Brady *et al.* (2017): 50%<br><br>Mihanović *et al.* (2021): 20%<br><br>de Sousa *et al.* (2018): 60%<br><br>Health status: Brady *et al.* (2017): BMI 30<br><br>Mihanović *et al.* (2021): BMI 20<br><br>de Sousa *et al.* (2018): Not reported	Same number of reprocessing cycles:<br><br>Brady *et al.* (2017): 1<br><br>Mihanović *et al.* (2021): 1<br><br>de Sousa *et al.* (2018): 1
