# Supplementary material for: Safety, cost and environmental impact of reprocessing low and moderate risk single-use medical devices: a systematic review
Source: GMS Hyg Infect Control. 2026 Jan 26;21:Doc08. doi: 10.3205/dgkh000617 (PMC12921675; doi:10.3205/dgkh000617)
Supplement: Grading of recommendations, assessment, development and evaluations [file HIC-21-08-s-005.pdf]

Attachment 5

Grading of recommendations, assessment, development and evaluations

Table S1 Grading of Recommendations, Assessment, Development and Evaluations (GRADE) table with explanations of ratings for individual domains

Outcome	A priori ranking	Downgrade for					Upgrade for			Final grade
		Risk of bias	Inconsistency	Indirectness	Imprecision	Publication bias	Large consistent effect	Dose response	Confounders only reducing size of effect	
External fixator devices										
Pin tract infections	Low:<br><br>One RCT and one observational study.	Serious limitation – downgrade by one:<br><br>Results based on studies of high and critical risk of bias (each in one domain).	Serious limitation – downgrade by one: Varied point estimates and overlapping confidence intervals. Can't explain differences e.g., whether differences are due to population, intervention, or outcomes and/or to non-reporting of same in the Dirschl and Smith study	Very serious limitation – downgrade by two: No details of the study population reported in the study by Dirschl and Smith (50% of all studies contributing data). The intervention context differed between studies - Dirschl and Smith compared several device brands whereas Sung *et al.* examined a single brand, reprocessing was undertaken in different reprocessing locations and Dirschl and Smith did not report findings by the number of	Very serious limitation – downgrade by two:<br><br>Wide confidence intervals the study by Dirschl and Smith (50% of all studies contributing data), both with appreciable benefit and harm. Both studies were likely underpowered based on Sung *et al.* assessment "Power analysis indicates that minimum of 1,600 patients would be necessary to demonstrate	No serious limitations – no downgrade:<br><br>Our search is comprehensive. Our findings are largely positive and unadjusted.	No upgrade:<br><br>Inconsistent findings.	No upgrade: Dose-response not applicable.	No upgrade: No adjustment for confounders.	Very low

Outcome	A priori ranking	Downgrade for					Upgrade for			Final grade
		Risk of bias	Inconsistency	Indirectness	Imprecision	Publication bias	Large consistent effect	Dose response	Confounders only reducing size of effect	
				reprocessing cycles (i.e. one and tow). Outcome reporting time was not reported in the study by Dirschl and Smith.	equivalence in the most common complication which was pin tract infections."					
Reoperations	Low:<br><br>One observational study.	Serious limitation - downgrade by one:<br><br>Results based on study of critical risk of bias (in one domain)	Serious limitation - downgrade by one:<br><br>Result based on one study	Serious limitation - downgrade by one:<br><br>No details of the study population, several device brands, and indirect comparison (no. reprocessing cycles not disaggregated i.e. between 1 and 2).	Very serious limitation - downgrade by two:<br><br>Wide confidence interval with appreciable benefit and harm, likely underpowered	No serious limitations - no downgrade:<br><br>Our search is comprehensive; findings largely positive and unadjusted	No upgrade:<br><br>One study	No upgrade:<br><br>Dose response N/A	No upgrade:<br><br>No adjustment for confounding	Very low
Endoscopic and laparoscopic devices										

Outcome	A priori ranking	Downgrade for					Upgrade for			Final grade
		Risk of bias	Inconsistency	Indirectness	Imprecision	Publication bias	Large consistent effect	Dose response	Confounders only reducing size of effect	
Postoperative complications (complications and/or reoperations)	Low: Two of three studies are observational	Serious limitation – downgrade by one:<br><br>Results based on studies receiving risk of bias scores of some concerns, moderate concerns and serious concerns in one or more domains.	No serious limitations – no downgrade:<br><br>Similar point estimates and overlapping (sometimes wide confidence intervals). Meta-analysis not undertaken due to differences in outcome definition.	Serious limitation – downgrade by one:<br><br>Differences in study population, study procedures and reprocessing location in the Mihanovic *et al.* study compared to others, device brands were not reported in two studies	Serious limitation – downgrade by one:<br><br>Wide confidence interval in 2/3 studies, confidence intervals in all studies reported appreciable benefit and harm. No power calculation undertaken in any study. Small sample sizes in 2/3 studies	No serious limitations – no downgrade:<br><br>Our search is comprehensive. Our findings are unadjusted.	No upgrade:<br><br>Consistent findings, potential for confounders.	No upgrade:<br><br>Dose-response not applicable.	No upgrade:<br><br>No adjustment for confounders.	Very low
Hospitalisation costs (indirect)	Low:<br><br>One observational study	Serious limitation – downgrade by one:<br><br>Result based on study of serious risk of bias concerns	No serious limitations – no downgrade:<br><br>Relatively narrow interquartile range	No serious limitations – no downgrade:<br><br>Comparable population for intervention and comparison groups	Serious limitation – downgrade by one:<br><br>Wide confidence interval and small sample	No serious limitations – no downgrade:<br><br>Our search is comprehensive. Our findings are unadjusted.	No upgrade:<br><br>One study	No upgrade:<br><br>Dose-response not applicable	No upgrade: No adjustment for confounders	Very low
